# Melatonin Pretreated Blastocysts along with Calcitonin Administration Improved Implantation by Upregulation of Heparin Binding-Epidermal Growth Factor Expression in Murine Endometrium

**DOI:** 10.22074/cellj.2018.4737

**Published:** 2017-11-04

**Authors:** Fatemeh Moghani-Ghoroghi, Ghazaleh Moshkdanian, Mojtaba Sehat, Seyed Noureddin Nematollahi-Mahani, Iraj Ragerdi-Kashani, Parichehr Pasbakhsh

**Affiliations:** 1 Department of Anatomy, School of Medicine, Tehran University of Medical Sciences, Tehran, Iran; 2 Anatomical Science Research Center, Kashan University of Medical Sciences, Kashan, Iran; 3 Department of Social Medicine, Faculty of Medicine, Kashan University of Medical Sciences, Kashan, Iran; 4 Department of Anatomy, Afzalipour School of Medicine, Kerman University of Medical Sciences, Kerman, Iran

**Keywords:** Blastocyst, Calcitonin, HB-EGF, Implantation, Melatonin

## Abstract

**Objective:**

Implantation failure is an obstacle in assisted reproduction techniques (ART). Calcitonin is a molecules involved in uterine receptivity and embryo implantation. Melatonin can promote embryo quality and improve implantation. This study examines the effect of pretreatment of blastocysts with melatonin and calcitonin on heparin binding-epidermal growth factor (HB-EGF) expression in murine endometrium.

**Materials and Methods:**

In this experimental study, we collected 2-cell embryos from the oviducts of 1.5 day pregnant NMRI mice. Embryos were cultured to the blastocyst in G^TM^ medium with or without 10^-9^ M melatonin. Pregnant and pseudo-pregnant mice received intraperitoneal (IP) injections of 2 IU calcitonin. After 24 hours, we transferred the cultured blastocysts into the uteri of pseudo-pregnant mice. Two days later, implantation sites were counted and we assessed the levels of HB-EGF mRNA and protein in the uteri of naturally pregnant and pseudo-pregnant mice by quantitative real-time polymerase chain reaction (qRT-PCR) and Western blot. Statistical analysis was performed with one-way ANOVA followed by the Tukey post hoc test. P<0.05 was considered statistically significant.

**Results:**

Melatonin pretreatment of blastocysts along with calcitonin administration significantly increased HB-EGF mRNA and protein (P<0.001) in the endometrium of pseudo-pregnant mice. Administration of calcitonin in naturally pregnant mice significantly increased HB-EGF mRNA and protein levels (P<0.001). Compared with the control group (2.6 ± 0.5), the average number of implantation sites in the melatonin group (4.6 ± 0.5, P<0.05) and calcitonin group (7 ± 1, P<0.001) significantly increased. There was a significant increase in implantation sites in the combined melatonin and calcitonin group (8.6 ± 0.5, P<0.001). Calcitonin significantly enhanced calcitonin receptor mRNA (P<0.001) and protein (P<0.05) in the uteri of naturally pregnant and pseudo-pregnant mice.

**Conclusion:**

Melatonin pretreated blastocysts along with calcitonin increased HB-EGF expression in the uteri of pseudo- pregnant mice. Calcitonin administration upregulated HB-EGF in uteri of naturally pregnant mice.

## Introduction

Infertility is one of the main problems amongst reproductive age couples. Approximately 12-16% of European and Asian couples suffer from infertility (1). Although the majority of these couples use assisted reproduction techniques (ART), the pregnancy rates remain low (2, 3). Unsuccessful embryo implantation is suggested to be the most important cause of this low pregnancy rate (4, 5). Less than 19% of transferred embryos can implant and develop to the delivery of a live neonate (6). Successful implantation and pregnancy occur by the simultaneous presence of a well-developed blastocyst and a receptive uterine endometrium. Alongside the deficiency of uterine receptivity, a low quality embryo may result in implantation failure in ART (7, 8). Despite the use of some medications to prevent implantation failure such as progesterone (9), leukemia inhibitory factor (LIF) (10), heparin, and aspirin, it has been reported that unfortunately these treatments do not have a valuable effect on uterine receptivity (10-12). Therefore, improving embryo quality and endometrial receptivity is a main concern in ART (13).

Embryo implantation is regulated by different types of growth factors, cytokines, and hormones (14, 15). Calcitonin is one of the factors that expresses in the uterine epithelium during implantation (16). Suppression of calcitonin mRNA during the pre-implantation phase has been shown to considerably diminish the number of embryos in rats (17). Calcitonin supported trophoblastic outgrowth on human endometrial epithelial cells (EEC) (18). An indirect upregulation of heparin binding-epidermal growth factor (HB-EGF) has been reported after calcitonin administration (13). HB-EGF is a transmembrane protein expressed at the site of apposition in the endometrium and has a critical role in attachment and the invasion processes of implantation (19, 20). Initially, studies have reported that HB-EGF was a key factor of embryo implantation in mice and rats (21). Researchers have reported that HB-EGF expressed in human endometrium. Binding of HB-EGF to its receptors triggers signaling cascades which develop endometrial receptivity and are essential for implantation (20). It has been reported that HB-EGF triggers hatching of blastocysts from the zona pellucida (19). Thus, HB-EGF is a crucial molecule in implantation (22).

On the other hand, reactive oxygen species (ROS) is produced through the embryo culture (23) which causes cell damage, apoptosis, and alterations in gene expression (24). Melatonin (N-acetyl-5- methoxytryptamine) is an effective free radical scavenger and antioxidant compared with vitamins C and E (23). Ishizuka et al. (25) have observed an increase in successful *in vitro* fertilization (IVF) after melatonin administration. Tian et al. (26) also reported a more developed and hatching blastocyst rate, as well as additional cell numbers in culture medium that contained 10^-9^ M melatonin. It is important to have a high quality embryo and increased uterine receptivity to improve implantation rates. We have designed this study to investigate the level of HB-EGF expression in the uteri of mouse pseudo-pregnant foster mothers following transfer of melatonin pretreated blastocysts in combination with calcitonin injection.

## Materials and Methods

### Animals and embryo collection

In this experimental study, we purchased 60 female NMRI mice (30-35 g), about 6 to 8-weeks old, from the Pharmacy Faculty of Tehran University of Medical Sciences (Tehran, Iran). All animal experiments were carried out according to the guidelines of the Iranian Council for Use and Care of Animals and approved by the Animal Research Ethical Committee of Tehran University of Medical Sciences (IR.TUMS. REC.1395.2884). The mice were housed in an airconditioned room under a 12 hour light: 12 hour dark cycle (7 am: 7 pm) with free access to food and water (13). In this study, we used naturally pregnant and pseudo-pregnant mice that were divided into 9 groupsthree groups of naturally pregnant mice and 6 groups of pseudop-regnant mice.

After two weeks of acclimation, female mice were superovulated by intraperitoneal (IP) injections of 5 IU pregnant mare serum gonadotropin (PMSG, Sigma, USA) followed by an IP injection of 5 IU human chorionic gonadotropin (hCG, Karma, Germany) 48 hours later (25). Then, the females were allowed to mate with fertile male NMRI mice overnight. The following day, the mice were examined for the presence of a vaginal plug; this date was designated as 0.5 days post coitum (dpc) (13). Mice with a vaginal plug were considered pregnant. We randomly divided these mice into 3 groups of 3 mice per group. At 2.5 dpc (27), the first group, pregnant mice+calcitonin (P+Cal) received 2 IU of calcitonin (Abcam, USA) (13). The second group, pregnant mice+normal saline (P+N.S) received normal saline. The third group, control or pregnant mice (P) received no treatment.

Mice that had a positive vaginal plug were sacrificed by cervical dislocation 46-48 hours after the hCG injection to collect the embryos. The 2-cell stage embryos were mechanically flushed with Ham’s F10 medium (Merck Millipore, USA) supplemented with BSA (4 mg/ml) pre-warmed in an incubator at 37˚C. Two-cell embryos were evaluated under a stereomicroscope (Nikon SMZ- 2T, Japan) and we randomly selected morphologically normal embryos (28) for further experiments.

### Embryo culture

A total of 10-15 normal embryos were cultured in 35- 50 μl microdrops of G-1TM (Vitrolife, Sweden) medium with or without 10^-9^ M melatonin (Sigma, USA) (26) under mineral oil (Sigma, USA) in a humidified incubator with 5% CO_2_ and 37˚C. The following day embryos were transferred to 35-50 μl microdrops of G-2TM (Vitrolife, Sweden) medium with or without 10^-9^ M melatonin. We observed, 48 to 72 hours after initiation of culture, embryos at the early blastocyst, late blastocyst, and hatching blastocyst stages. Welldeveloped blastocysts were randomly selected to transfer to the pseudo-pregnant foster mothers.

### Embryo transfer and *in vivo* tests

**Fig.1:**
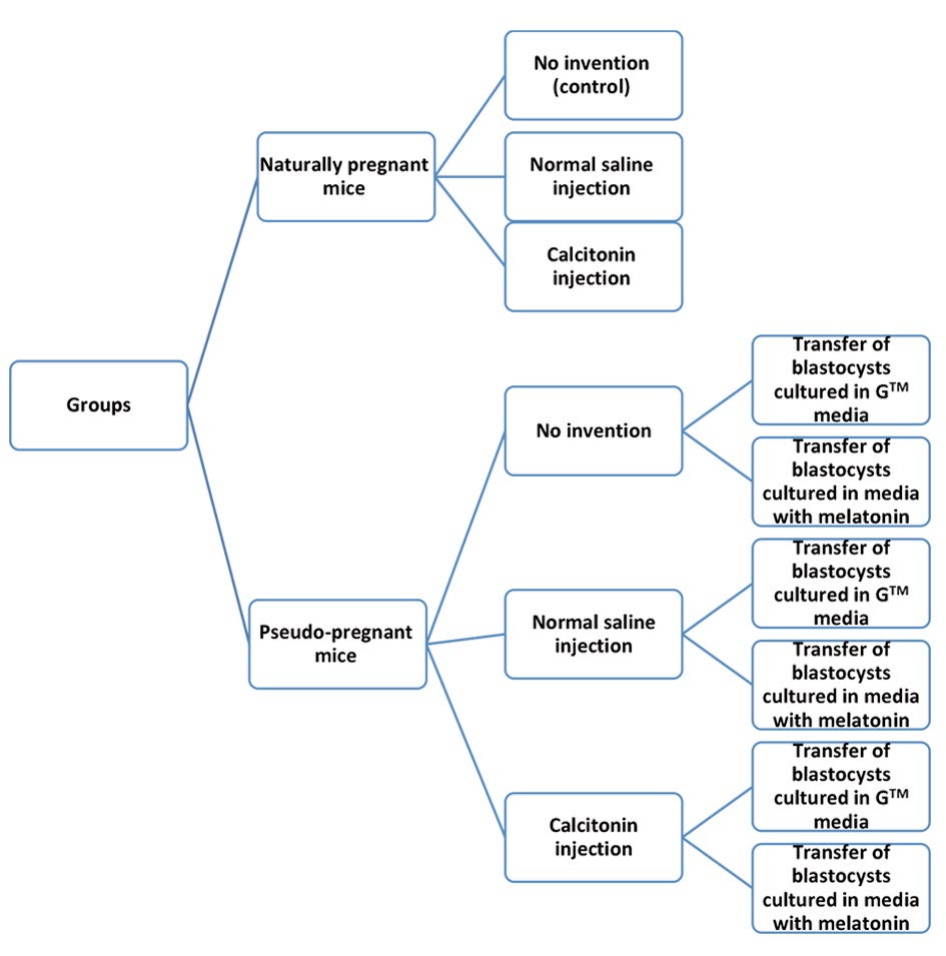
Summary of the different experimental groups.

The 6 to 8-week-old virgin female NMRI mice were used as pseudo-pregnant foster mothers (or embryo recipients). For producing pseudo-pregnant mice as recipients or foster mothers, after induction of superovulation (as mentioned earlier) the mice were mated with vasectomized male mice of the same race (29). Mice that had a positive vaginal plug were randomly divided into 6 groups of 3 mice per group. The first and second groups consisted of pseudo-pregnant foster mothers that received transferred blastocyst cultured in G media as the control group (Pseudo- P/G) or media supplemented with 10^-9^ M melatonin (Pseudo-P/M). The third and fourth groups consisted of pseudo-pregnant foster mothers who received an IP injection of normal saline and blastocysts cultured in G media (Pseudo-P+N.S/G) or media supplemented with melatonin (Pseudo-P+N.S/M). In the fifth and sixth groups, pseudo-pregnant foster mothers received IP injections of 2 IU calcitonin 24 hours before embryo transfer and blastocysts cultured in G media (Pseudo- P+Cal/G) or media supplemented with melatonin (Pseudo-P+Cal/M). Figure 1 summarizes the dif ferent groups.

Under general anesthesia, we made a small hole on the left uterine horn a few millimeters further from the utero-tubal junction. Then, we used IVF Pasteur pipets (GmbH, Germany) to transfer 10 embryos per mouse (30) through the hole into the left uterine lumen of the recipients (31). After surgery the mice were allowed to recover in a clean cage with careful handling to prevent stressful conditions for the pregnant mice. The mice received intravenous administration of 0.1- 0.2 ml Chicago sky blue dye (Santa Cruz, CA, USA, 1% in saline) 48 hours after embryo transfer (32). After 30 minutes, the mice were killed. Blue bands in each uterus were considered to be the implantation sites, which we counted and compared them in the different groups.

### Differential staining of blastocysts

We randomly chose expanded blastocysts for cell counting analysis. Blastocysts were removed from the culture media and placed in Ham’s F10 medium supplemented with 1% Triton X-100 and 100 μg/ml propidium iodide (PI, Sigma, USA) for approximately 30-40 seconds. Then blastocysts were incubated in droplets that contained 25 μg/ml bisbenzimide (Hoechst, Sigma, USA), overnight at 4˚C in a dark chamber (29). The blastocysts were washed three times in phosphate-buffered saline (PBS) to remove residual dyes. Thereafter, embryos were mounted in a drop of glycerol on a microscope slide and covered by a coverslip. Samples were examined as soon as possible under fluorescent microscope (Olympus BX51TRF, Japan) equipped with a UV filter. The inner cell mass (ICM) nuclei were characterized with bisbenzamide (350-461 nm) and appeared blue, whereas the outer trophectoderm (TE) nuclei were recognized by the pink fluorescence of PI (535-617 nm). The ICM and TE cell numbers, and total cell numbers (TCN) were counted (33).

### RNA extraction and quantitative real-time polymerase chain reaction

Expression of *HB-EGF* and *calcitonin receptor* genes were assessed by quantitative real-time polymerase chain reaction (qRT-PCR) with TRIzol® reagent (Cinnagen, Iran). Total RNA was isolated from endometrial tissue, then mRNA (1 μg) was converted to cDNA via reverse transcription with an AccuPower® RocketScript™ RT PreMix kit (Bioneer Company). Specific primers along with cDNA and PCR reagents were placed into a real-time PCR machine (Applied Biosystems Step One, USA). The samples underwent an initial polymerase activation stage at 95˚C for 15 minutes, followed by denaturation at 95˚C for 15 seconds, annealing at 60˚C for 20 seconds, and elongation at 72˚C for 20 seconds. Finally, we used the ΔΔCt technique for relative quantification of the data and further normalization to *β-actin* and fold change compared to the control. The primers were designed with Gene Runner (version 3) and Primer Express (version 3.05) software. The designed primers were blasted in http://www.ncbi.nlm.nih.gov/BLAST/. Table 1 lists the nucleotide sequences of the primers.

**Table 1:** Primer Sequences for quantitative real-time polymerase chain reaction


Genes	Primer sequence (5ˊ-3ˊ)	Product size (bp)

HB-EGF	F: CCAGTTGCTACCCTGACTGG	136
	R: GAAGGGCTCACTCGATCCTG	
Calcitonin receptor	F: TAGTTAGTGCTCCTCGGGCT	116
	R: AGTACTCTCCTCGCCTTCGT	
β-actin	F: CCACCATGTACCCAGGCATT	253
	R: AGGGTGTAAAACGCAGCTCA	


HB-EGF; Heparin binding-epidermal growth factor.

### Western blot analysis

Western blot was performed to analyze HB-EGF and calcitonin receptor expressions at the protein levels. Defined proteins were acquired from frozen uterine tissue by homogenization. Proteins were separated by 12% sodium dodecyl sulfate polyacrylamide gel electrophoresis (SDS-PAGE). Proteins were fixed and stained by Kumasi blue dye to determine the protein position and concentration on blot and western set up. Then proteins transferred onto nitrocellulose membranes. The membranes were blocked in tris-buffered saline that contained 0.05% Tween-20 buffer (TBST) with 5% nonfat milk, and then incubated with anti-HB-EGF (Santa Cruz, CA, USA), anti-calcitonin receptor, and anti-β- actin antibody (Abcam, Germany, 1/100) overnight at 4˚C followed by a one hour incubation with horseradish peroxidase (HRP) secondary antibody. Immunoreactive bands were envisioned by enhanced chemiluminescence. Finally, specific bands were quantified using Total Lab Quant analysis software (Total Lab Limited, UK). We analyzed the expression ratio of proteins to β-actin.

### Statistical analysis

All experiments were performed in triplicate. The data are expressed as mean ± SD. To evaluate the statistical significance between different groups, we used one-way analysis of variance (ANOVA) followed by Tukey’s and Tamhane’s post hoc tests and the independent-samples t test, using SPSS 16. P<0.05 was considered statistically significant.

## Results

### Effect of blastocysts pretreated with melatonin on heparin binding-epidermal growth factor expression

**Fig.2:**
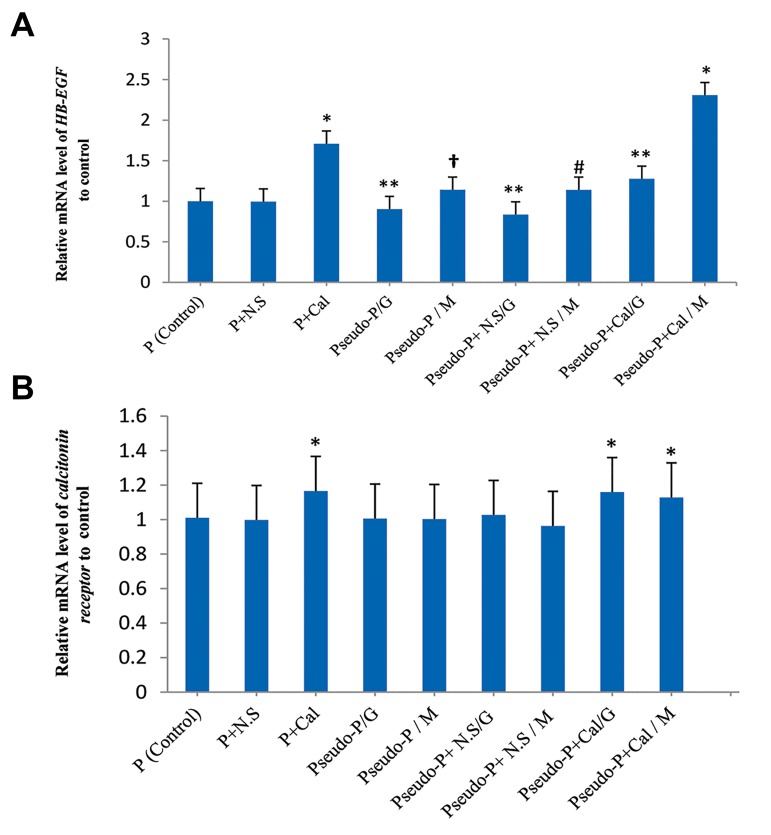
Quantitative real-time polymerase chain reaction (QRT-PCR) analysis of *HB-EGF* and *calcitonin receptor* mRNA. A. The graph shows that the highest *HB-EGF* expression was observed in the Pseudo-P+Cal/M group. Also a significant higher expression was observed in the melatonin treated groups. In addition, calcitonin increased *HB-EGF* mRNA in endometrial tissues of naturally pregnant mice. Error bars represent means ± SD (n=3). *; P<0.001 vs. control and all experimental groups, **; P<0.01 vs. control and all experimental groups, #; P<0.001 vs. control and all experimental groups, except the Pseudo-P/M group, †; P<0.01 vs. control and all experimental groups except the Pseudo-P+N.S/M group and B. The graph shows that the mRNA level of *calcitonin receptor* in the calcitonin groups was higher than the other groups. *; P<0.001 vs. control and all experimental groups. N.S; Normal Saline, Cal; Calcitonin, Pseudo-P; Pseudo-Pregnant, M; Media contain Melatonin, and G; GTM Media.

**Fig.3:**
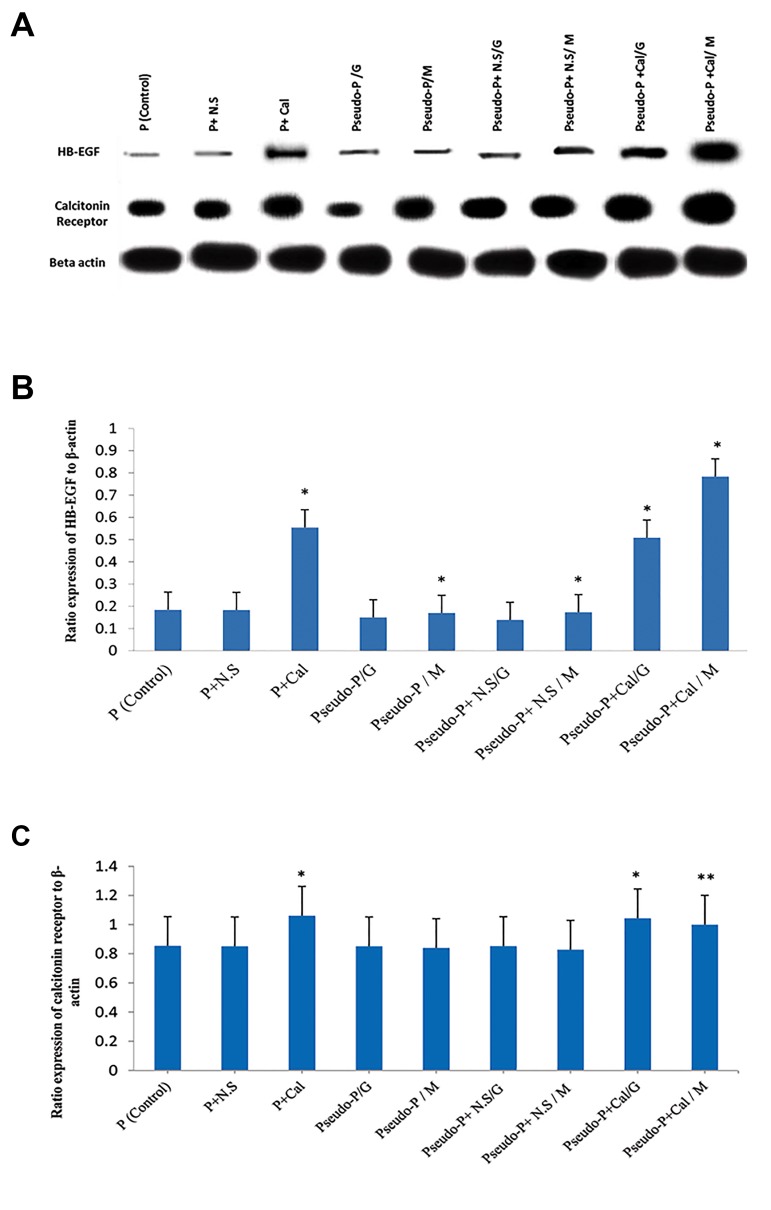
Western blot analysis of HB-EGF and calcitonin receptor. A. Western blot bands of mentioned genes in different groups, B. As the graph shows, the Pseudo-P+Cal/M group had the highest expression of HB-EGF. The P+Cal and Pseudo-P+Cal/G groups had significantly higher expression. Data are shown as mean ± SD (n=3). *; P<0.001 vs. the control and all experimental groups, and C. Graph shows that Calcitonin receptor expression was higher in the calcitonin groups. **; P<0.01 vs. the control and all experimental groups, except the P+Cal and Pseudo-P+Cal/G groups. *; P<0.05 vs. the control and all experimental groups, except the Pseudo-P+Cal/M group. N.S; Normal saline, Cal; Calcitonin, Pseudo-P; Pseudo-pregnant, M; Media contain melatonin, and G; GTM media.

We sought to explore the possible effects of melatonin on gene expression in the murine endometrium by transferring blastocysts pretreated with melatonin into the uteri of pseudopregnant foster mothers. QRT-PCR showed that blastocysts pretreated with 10^-9^ M melatonin induced and upregulated *HB-EGF* expression in the endometrium. The Pseudo-P/M, Pseudo-P+N.S/M, and Pseudo-P+Cal/M groups that received the melatonin pretreated blastocysts had significantly greater *HB-EGF* mRNA compared to the Pseudo-P/G (P<0.01), Pseudo-P+N.S/G (P<0.001), and Pseudo-P+Cal/G (P<0.001) groups (Fig .2). Western blot analysis confirmed the qRT-PCR results. There was a significant increase in HB-EGF protein in the Pseudo-P/M, Pseudo-P+N.S/M, and Pseudo-P+Cal/M groups compared to the Pseudo-P/G, Pseudo-P+N.S/G, and Pseudo-P+Cal/G groups (P<0.001, Fig .3A, B).

### The effect of calcitonin and melatonin pretreated blastocysts on heparin binding-epidermal growth factor expression

The results show that transfer of melatonin pretreated blastocysts to the uteri of pseudo-pregnant mice that received a single dose of calcitonin had significantly upregulated mRNA expression of *HB-EGF* compared with the control and other experimental groups (P<0.001, Fig .2). At the protein level, Western blot analysis confirmed these results. A significant increase existed in HB-EGF protein levels in the Pseudo-P+Cal/M group (P<0.001, Fig .3A, B). In addition, our results showed that administration of calcitonin augmented mRNA levels of HB-EGF in endometrial tissues of naturally pregnant mice in the P-Cal group (P<0.001) and pseudo-pregnant foster mother mice following blastocyst transfer for the Pseudo- P+Cal/G (P<0.01) and Pseudo-P+Cal/M (P<0.001) groups (Fig .2A). Western blot analysis confirmed the increased *HB-EGF* expression in the endometrial tissues of the P-Cal, Pseudo-P+Cal/G, and Pseudo-P+Cal/M groups. There was significantly greater HB-EGF protein in the endometria of the groups that received calcitonin compared to the other groups (P<0.001, Fig .3A, B).

### Calcitonin upregulates expression of the calcitonin receptor in murine endometrium

Our data demonstrated that calcitonin receptor expression increased at the mRNA level in the P-Cal, Pseudo-P+Cal/G, and Pseudo-P+Cal/M groups (P<0.001, Fig .2B). Western blot results showed a significant increase in protein level of the calcitonin receptor in the P-Cal, Pseudo-P+Cal/G, and Pseudo-P+Cal/M groups treated with calcitonin (P<0.001, Fig .3A, C).

### Differential blastocyst staining

**Fig.4:**
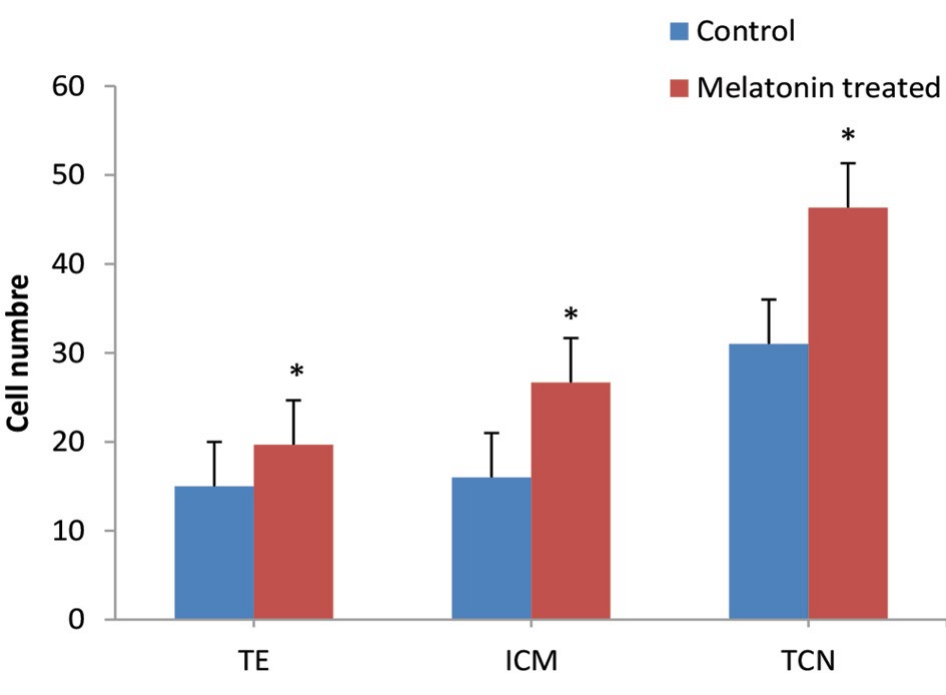
Effects of melatonin on blastocyst cell numbers. Melatonin significantly increased the inner cell mass (ICM), trophectoderm (TE) cell number, and total cell number (TCN) compared to the control group. Data are shown as mean ± SD. (n=3). *; P<0.05 vs. control.

We examined the effect of melatonin on embryo development by randomly staining the blastocysts with PI/Hoechst. The ICM, TE, and TCN were counted under a fluorescent microscope. As the results show, there was a significant increase in ICM, TE cell number, and TCN in melatonin pretreated groups compared with the control group (P<0.05, Fig .4).

### Pretreatment of blastocysts with melatonin along with administration of calcitonin increased implantation of blastocysts in vivo

**Fig.5:**
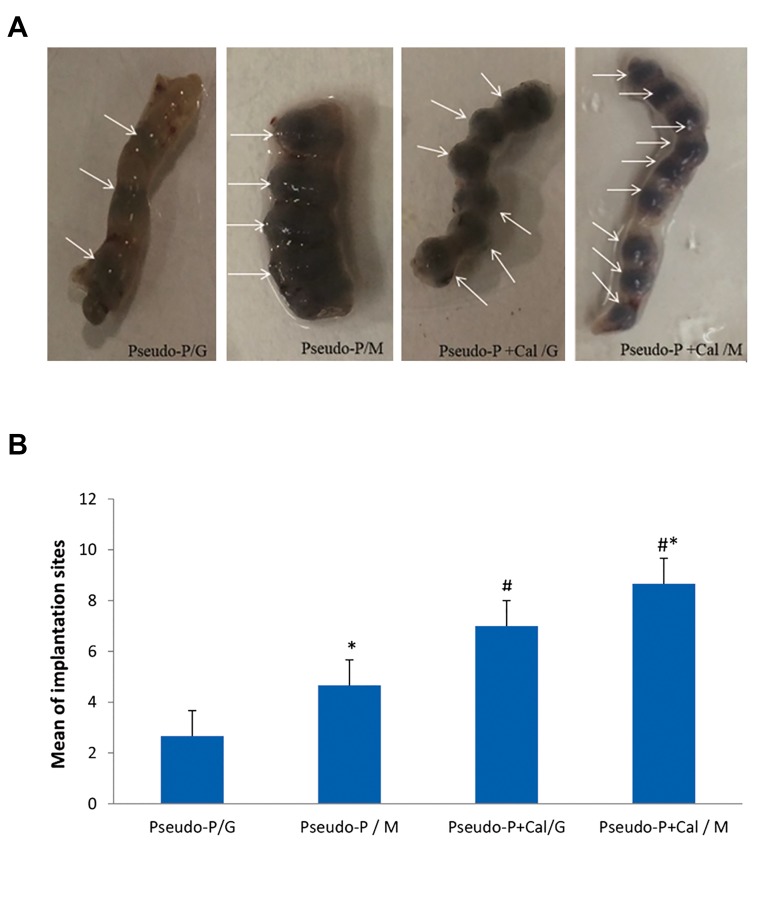
Effect of melatonin pretreated blastocysts along with administration of calcitonin on implantation of mouse blastocysts. The implantation sites were counted and compared in different groups. A. The arrows indicate implantation sites in the uterus and B. The average number of implantation sites. Data are shown as mean ± SD (n=3). *; P<0.05 vs. Pseudo-P+Cal/G and #; P<0.001 vs. Pseudo-p/G. N.S; Normal saline, Cal; Calcitonin, Pseudo-P; Pseudo-pregnant, M; Media contain melatonin, and G; GTM media.

According to the results, calcitonin enabled the endometrium to be more receptive for blastocysts to attach and implant. We checked the implantation sites 48 hours after the embryo transfer (Fig .5A). We observed that Pseudo- P+Cal/M group had the highest number of implantation site (8.6 ± 0.5, Fig .5B). The melatonin pretreated groups (Pseudo- P/M and Pseudo-P+Cal/M) had significantly more average number of implantation sites compared to the Pseudo-P/G (P<0.001) and Pseudo-P+Cal/G (P<0.05) groups. Compared with the Pseudo-P/G group (2.6 ± 0.5), we observed an increased average number of implantation sites in the Pseudo- P+Cal/G (7 ± 1) and Pseudo-P+Cal/M (8.3 ± 0.5) groups (both P<0.001, Fig .5B).

## Discussion

ART procedures intend to overcome infertility and result in a greater pregnancy rate (13, 34). Although ART technique has been developed broadly, implantation failure is still one of the main obstacles for ART (13). *HB-EGF* is one of the most important implantation genes in both humans and mice (22). Studies show that *HB-EGF^-/-^* female mice are sub-fertile (35). Previous studies have stated that active blastocysts are the main inducers of *HB-EGF* expression (21). Embryo quality may define the state of activity of a blastocyst (36). It has been suggested that the presence of an active blastocyst is needed to stimulate implantation (22). It is well known that different environmental factors such as an *in vitro* culture system and conditions, a high oxygen concentration (37, 38) and pH fluctuations (39) can produce more oxidative stress, which is harmful for early embryonic development (23, 40, 41) and affect embryo quality and viability (42). They can also change embryo gene expression (37,43, 44). Recently melatonin, a free radical scavenger and antioxidant, has been broadly used as a protective agent in the embryo culture (23, 45).

In the present study, we investigated the effect of melatonin pretreated blastocysts on HB-EGF expression in the uteri of pseudo-pregnant mice. Our results showed that pretreatment with melatonin increased expression of HB-EGF mRNA and protein after blastocyst transfer. Consistent with the current study findings, a recent study reported that pregnant mice injected with melatonin had increased HB-EGF expression in their endometrium and increased blastocyst activation *in vivo* (46). Morphological assessment of the blastocysts demonstrated that 10^-9^ M melatonin significantly enhanced TCN, ICM, and TE cells. Previous studies reported the same findings (26). As mentioned earlier, in addition to a high quality embryo, a receptive uterine is essential for successful implantation (47, 48). In recent years, various biological factors involved in endometrial receptivity have been identified (49). Among these, calcitonin is a well-known putative implantation gene (18) that plays a crucial role in uterine receptivity during implantation (34, 49, 50). In an *in vitro* EEC model, calcitonin has been shown to increase the outgrowth of trophoblasts on a human EEC monolayer (18). These results suggest that exogenous calcitonin may promote the competence of uterine receptivity and embryo implantation (13).

We investigated the effect of melatonin pretreated blastocyst and calcitonin administration on HB-EGF expression in uterine of pseudo-pregnant mice, as an in vivo model. Our data demonstrated that calcitonin administration significantly increased HB-EGF mRNA and protein in naturally pregnant mice and in pseudopregnant mice after blastocyst transfer, which supported the results of a previous study where calcitonin indirectly up regulated *HB-EGF* mRNA expression in EECs (13). Transfer of melatonin pretreated blastocysts into pseudopregnant mice that received calcitonin significantly increased HB-EGF mRNA and protein.

Taken together, these findings proposed that melatonin pretreated blastocysts along with administration of calcitonin could increase HB-EGF expression, a molecule associated with endometrial receptivity and embryo implantation. Consistent with previous reports, our results showed a significant increase in implantation rate in the melatonin (23) and calcitonin (13) groups. The implantation rate significantly increased in pseudopregnant recipients that simultaneously received melatonin pretreated blastocysts and calcitonin. Calcitonin acts through binding to its cell surface receptor, a seven transmembrane G-protein-coupled receptor, to stimulate several signaling pathways that include the adenylyl cyclase and phospholipase C pathways. Adenylyl cyclase activation results in an increase in intracellular cyclic adenosine monophosphate (cAMP), which stimulates protein kinase A (PKA). In addition, inositol triphosphate is produced through phospholipase C activation which results in release of Ca^2+^ from intracellular stores (18).

Previous studies have reported that melatonin can promote blastocyst activation both *in vivo* and *in vitro* through changes in expression of some important embryo development and implantation-related genes (37, 46). A number of these genes involved in Ca^2+^ signaling and the inositol 1, 4, 5-trisphosphate pathway significantly upregulates in activated blastocysts. Studies have revealed that blastocyst implantation can be regulated by Ca^2+^ signaling (51). Therefore, possibly melatonin and calcitonin can upregulate HB-EGF expression through a similar mechanism.

Our results have demonstrated that calcitonin receptor significantly upregulated in murine endometrium after calcitonin administration. To our knowledge, there is little information about the effect of calcitonin on alteration of the calcitonin receptor in the uterus. Previous studies have reported that increased *calcitonin receptor* mRNA in blastocysts and calcitonin levels in the uterus occur simultaneously (52). Based on our data and reports from other studies, it can be suggested that calcitonin may induce calcitonin receptor expression in blastocysts and uteri. However, the current study results contrasted studies which have reported downregulation of the calcitonin receptor in osteoclasts after calcitonin administration (53). Additional research is required to explore the mechanism of an increased calcitonin receptor in endometrium via calcitonin administration. Given that HB-EGF is a crucial regulator of implantation (22), endometrial receptivity and embryo transfer programs may be promoted by pretreatment of blastocysts with melatonin and calcitonin administration. Additional research is required to prove the efficacy of blastocysts pretreated with melatonin and calcitonin in improving HB-EGF expression in endometrium and in embryo implantation during ART.

## Conclusion

This study provided evidence that pretreatment of blastocysts with 10^-9^ M melatonin and administration of calcitonin to naturally pregnant and pseudo–pregnant mice could enhance HB-EGF expression, a critical molecule associated with endometrial receptivity and embryo implantation. Calcitonin might increase calcitonin receptor expression in murine endometrium.
